# Peculiarities of Phosphatidylserine Externalization by Nano- and Microsecond Electric Pulses

**DOI:** 10.1007/s00232-026-00384-5

**Published:** 2026-06-04

**Authors:** Mantas Silkunas, Alayna Enos, Iurii Semenov, Giedre Silkuniene, Eleni Zivla, Olga N. Pakhomova, Andrei G. Pakhomov

**Affiliations:** Frank Reidy Research Center for Bioelectrics, Old Dominion University, 4211 Monarch Way, Suite 300, Norfolk, VA 23508 USA

**Keywords:** Electroporation, Electropermeabilization, Phosphatidylserine, Extracellular vesicles, Shedding vesicles, Membrane repair

## Abstract

Phosphatidylserine (PS) externalization is a hallmark of apoptosis but can also be triggered within seconds by pulsed electric fields (PEFs). Whether PS reaches the outer leaflet by lateral migration through electropores or indirectly through Ca^2+^-dependent signaling and scramblase activation remains debated. We used total internal reflection fluorescence (TIRF) microscopy with Annexin V-Alexa Fluor 568 and/or bovine lactadherin-FITC to resolve PS externalization in HEK293 cells placed on an indium tin oxide (ITO) transparent electrode. PEF exposures produced discrete PS-positive puncta (“freckles”) within ~ 10 s after a 20-µs pulse (1.2–2.5 kV/cm) or with a ~ 1-min delay after a 400-ns pulse (9.3–13.7 kV/cm). Their number gradually increased, reaching up to 80–120 freckles per 100 µm^2^ of cell membrane area within 5 min. Freckles were round, brightest in the center, and well described by a Gaussian intensity profile, with a consistent full width at half maximum (FWHM) of ~ 0.35 µm despite up to ~ 50-fold differences in peak intensity. Freckle formation was inhibited in Ca^2+^-free medium but could be partially restored by applying 20-µs PEFs at higher strengths. Many freckles moved laterally and axially in an apparently stochastic manner, whereas others remained spatially confined. The uniform size and shape of freckles and their mobility patterns suggested that they are PS-positive vesicles rather than PS membrane patches. Further analyses identified freckles as extracellular vesicles of ~ 200-nm estimated diameter whose formation is triggered by applying PEFs at intensities above the electroporation threshold.

**Graphical Abstract:**

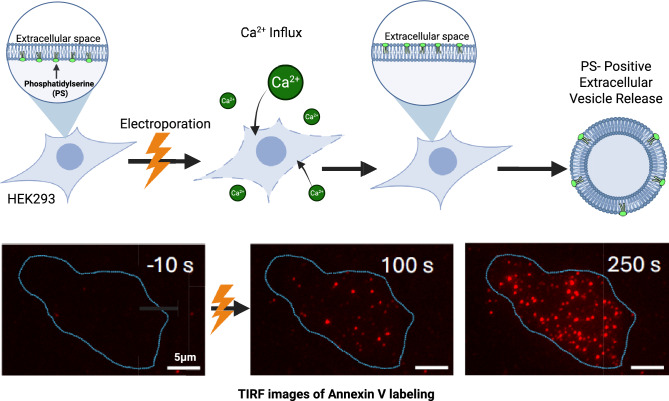

## Introduction

Phosphatidylserine (PS) is normally confined to the cytoplasmic leaflet of the plasma membrane by ATP-dependent lipid transporters. Loss of this lipid asymmetry and PS translocation to the cell surface is a key signal in physiology and pathology, including recognition and clearance of apoptotic cells, regulation of coagulation, and activation of immune responses (Copic et al. 2023; Nagata et al. 2020; Segawa and Nagata 2015; van Engeland et al. 1998; Yoo and Kim 2026). In live-cell experiments, surface PS is commonly detected by fluorescent PS-binding proteins such as Annexin V (Ca^2+^-dependent) and lactadherin (also known as MFG-E8; Ca^2+^-independent)(Kay and Grinstein 2011; Shi et al. 2006; Waehrens et al. 2009). PS detection is widely used as an operational marker of apoptotic commitment (Segawa and Nagata 2015; van Engeland et al. 1998).

Externalization of PS is also a well-established consequence of electroporation (Beebe et al. 2004, 2002; Muratori et al. 2017; Pakhomov et al. 2009; Roth et al. 2013; Vernier et al. 2006a, 2004a, 2004b; Vincelette et al. 2013). Intense pulsed electric fields (PEFs) transiently permeabilize the plasma membrane, enabling ionic fluxes and initiating Ca^2+^-dependent signaling. Early reports of rapid and extensive PS externalization after nanosecond PEFs (nsPEFs) interpreted it as a hallmark of apoptosis and viewed as supporting apoptosis as a dominant outcome of nsPEF treatments (Beebe et al. 2004, 2002; Hall et al. 2005). Subsequent work challenged this interpretation by showing that PS externalization can occur within seconds, a timescale incompatible with an apoptotic program (Pakhomov et al. 2009; Vernier et al. 2006a, 2004b, 2006b) and is tightly linked to membrane permeabilization and Ca^2+^ entry (Roth et al. 2013; Vincelette et al. 2013). Ca^2+^ influx through electropores can activate phospholipid scramblases, rapidly collapsing lipid asymmetry and driving PS to the outer leaflet (Bevers et al. 1999; Nagata et al. 2020; Zhao et al. 1998). In parallel, electropores provide a continuous lipid-water interface that may enable Ca^2+^-independent lipid rearrangements by "lateral drift" of inner-leaflet PS along the pore wall to the outer membrane surface (Vernier et al. 2006a, 2004b, 2006b; Vincelette et al. 2013). PS is negatively charged; hence, another hypothesized mechanism of externalization driven by strong electric fields is its translocation towards the anode through nanometer-scale defects formed by membrane blebbing (Tekle et al. 2008).

We have recently demonstrated that individual electropores can persist for hundreds of milliseconds (Silkunas et al. 2024) and even tens of seconds (Silkunas et al. 2023), in stark contrast to molecular models that suggested pore annihilation within nano- to microseconds after PEF is turned off (Kotnik et al. 2019; Levine and Vernier 2010; Rems et al. 2019; Tarek 2005). This discrepancy could be explained if long-lived electropores form in membrane proteins rather than in lipids (Kotnik et al. 2019; Marracino et al. 2018; Rems et al. 2020; Ruiz-Fernandez et al. 2021; Silkuniene et al. 2023a, 2023b). PS externalization by lateral diffusion is possible only in lipid pores and will not happen in hypothesized proteinaceous electropores. Accordingly, the original aim of this study was to test if the pattern of PS externalization is consistent with the lateral diffusion mechanism (colocalization with pores and lateral spread from the initial foci), in order to confirm or negate the lipid nature of electropores. Instead, we observed a distinctive punctate signature of PS externalization, manifested by the formation of numerous submicron PS-positive spots (“freckles”) with highly uniform apparent size and variable mobility. We analyzed the time course of their emergence, brightness, mobility, and Ca^2^⁺ dependence, and found that these features are most consistent with extracellular PS-positive extracellular vesicles shed from the cells. Although this outcome did not resolve whether electropores are lipidic or proteinaceous, it identified PS externalization via vesicle shedding as a previously unknown PEF response with potential implications for medical applications such as gene electrotransfer and tumor ablation.

## Materials and Methods

### Cells and Media

Human embryonic kidney cells (HEK293; American Type Culture Collection, Manassas, VA) were propagated at 37 °C in a humidified incubator with 5% CO_2_ in Eagle’s Minimum Essential Medium (EMEM; Thermo Fisher Scientific, Waltham, MA) supplemented with 10% fetal bovine serum (Atlanta Biologicals, Norcross, GA), 100 U/mL penicillin, and 0.1 mg/mL streptomycin (Gibco, Gaithersburg, MD).

Cells were seeded 20–24 h before experiments onto customized 35-mm glass-bottom dishes (MatTek, Ashland, MA) fitted with 15-mm diameter glass coverslips coated with indium tin oxide (ITO; sheet resistance 8–12 Ohm/sq, ~ 120-nm thick; Diamond Coatings Ltd., Halesowen, UK). ITO served as a transparent conductive electrode for PEF delivery (Michel et al. 2020; Silkunas et al. 2024, 2023).

Experiments were performed at room temperature in the physiological buffer containing (in mM): 140 NaCl, 5 KCl, 2 MgCl_2_, 2 CaCl_2_, 10 HEPES, and 10 glucose (pH 7.3 adjusted with NaOH; ~ 310 mOsm). For Ca^2+^-free conditions, CaCl_2_ was replaced with 2 mM Na-EGTA. The chemicals were obtained from Sigma-Aldrich (St. Louis, MO) and Thermo Fisher Scientific.

### Fluorescent Probes

Externalized PS was visualized with Annexin V-Alexa Fluor 568 (Thermo Fisher Scientific) and/or bovine lactadherin-FITC (Prolytix, Essex Junction, VT). Annexin V-Alexa Fluor 568 was supplied at 2 µg/mL in a HEPES/NaCl/EDTA buffer with 0.1% bovine serum. Lactadherin-FITC was supplied as a Tris-buffered saline solution with 1% bovine serum albumin and 0.02% sodium azide. Both dyes were diluted from the stocks 1:200 into the physiological buffer.

For Ca^2+^ imaging, cells were first incubated for 30 min at room temperature in the physiological buffer with 5 µM of Cal-520 AM (AAT Bioquest, Pleasanton, California). The cells were rinsed twice with the dye-free buffer and incubated for at least 10 min to allow dye de-esterification before the experiment.

In one set of experiments, the extracellular buffer was fluorescently labeled by adding 50 µg/mL of 10 kDa FITC–dextran (Sigma-Aldrich). FITC–dextran was employed as a physiologically inert tracer with no detectable entry into intact or PEF-treated cells.

TetraSpeck Fluorescent Microspheres of 110-nm size (Thermo Fisher Scientific) were utilized to measure the point spread function (PSF) of our imaging setup. They were diluted 1:2,000 in the physiological solution, dropped onto the same ITO coverslips that were used in cell experiments, allowed to settle down for ~ 10 min, and imaged in TIRF mode under 488 nm or 561 nm laser illumination.

### Pulsed Electric Field Exposure and Dosimetry

PEFs exposure and the electric field simulation methods were essentially the same as described and illustrated previously (Michel et al. 2020). A Petri dish containing cells on an ITO-coated coverslip was filled with the physiological buffer and mounted on the motorized stage of an Olympus IX83 inverted microscope (Olympus America, Center Valley, PA). A target cell was positioned at the center of the field of view. A tungsten rod electrode (100 µm diameter), fixed in the robotic micromanipulator (MPC-200, Sutter Instrument, Novato, CA) at 35° angle to the horizontal plane, was positioned with its lowest point above the target cell and precisely 100 µm above the coverslip. Unipolar, nearly rectangular 400-ns, 2-µs, or 20-µs pulses were applied between the rod and the ITO coating of the coverslip. In all but one sets of experiments, positive polarity was applied to the rod. 400-ns and 2-µs pulses were from a 6040 Universal Pulse Generator equipped with a 202H high-voltage module (Berkeley Nucleonics Corporation, San Rafael, CA). 20-µs pulses were from BNC Model 577 digital delay generator (Berkeley Nucleonics Corporation). Pulse shape and amplitude were monitored using a TEK 3052C oscilloscope with a TEK P6139A probe (Tektronix, Beaverton, OR).

Electric field strength was computed as described in detail earlier (Michel et al. 2020), and independently verified for the same geometry by finite-element modeling in Sim4Life 7.0.1.8169 (ZMT Zurich MedTech AG, Zurich, Switzerland). Immediately above the ITO surface, the field averaged 62 V/cm per 1 V applied within a 30 × 30 µm region beneath the electrode tip. This conversion coefficient was used throughout this study to obtain the electric field strength by multiplying it by the oscilloscope-measured pulse amplitude.

### Total Internal Fluorescence (TIRF) Microscopy and Image Analyses

Fluorescence imaging was performed on an Olympus IX83 inverted microscope equipped with a cellTIRF MITICO illuminator and a 100 × /1.50 NA UPLAPO100XOHR objective (Olympus America). All experiments were done in TIRF mode, with image acquisition controlled by Olympus cellSens 2.3 software. Excitation was provided by 488-nm and 561-nm lasers (Coherent, Santa Clara, CA) using a laser quad-band TRF89901-OL3 filter cube (Chroma Technology Corp, Bellows Falls, VT). Emission signals were separated by Lambda 10–2 filter wheel (Sutter Instrument) switching between 605/52 nm and 525/36 nm filters synchronously with the respective laser activation. Images were acquired with an Orca-Quest 2 Photon-Counting Gen-III qCMOS camera with 46 × 46 nm pixel size (Hamamatsu, Bridgewater, NJ) The acquisition of image stacks was synchronized with PEF delivery using an Olympus U-RTCE Real-Time Controller and cellSens interface.

Image stacks were analyzed in MetaMorph 7.7 (Molecular Devices, San Jose, CA), OlyVIA 3.1 (Olympus), or Fiji/ImageJ version 1.54p open-source platform. Cell outlines were drawn from transmitted-light images or the fluorescence background, and analysis was restricted to the basal membrane area within the TIRF field.

To characterize the overall dynamics and PEF dependence of freckle formation and their properties, a 10 × 10 µm square region of interest (ROI) was positioned over the area of the cell that visually contained the highest density of freckles. They were identified within this ROI automatically as objects < 5 µm in size and at least 100 grayscale levels above the local background. For each freckle, we computed (i) mean intensity (average grayscale value), (ii) peak intensity (maximum pixel value), and (iii) integrated intensity (sum of pixel values). For each cell, freckle mean and peak intensities were calculated by averaging the corresponding per-freckle values, and cumulative integrated intensity was calculated by summing per-freckle integrated intensities. Freckle variability was quantified as the standard deviation of per-freckle integrated intensities within the cell. These per-cell values were then averaged across independent experiments and plotted as mean ± s.e. Thus, error bars in all graphs characterize the variation across individual experiments (one cell per experiment) but not within them.

To measure the apparent freckle size, we extracted pixel intensity values along a 2-pixel-wide, 50-pixel-long line region of interest (ROI) centered on a freckle. Pixel values at the edges of the ROI (10 pixels at each end) were averaged and subtracted from all other values as background. The location of maximum intensity was taken as the freckle center and assigned the coordinate $X=0$. Pixel intensity along the line, $Y$(arbitrary units, a.u.), was approximated by a Gaussian:

$$Y=A\mathrm{e}\mathrm{x}\mathrm{p}\left(-4\mathrm{l}\mathrm{n}2{\hspace{0.17em}}\frac{{X}^{2}}{{W}^{2}}\right)$$

where *A* is the peak intensity (a.u.), *X* is the distance from the center (µm), and *W* is the full width at half maximum (FWHM, µm). All fits presented in this paper had high correlation coefficients $\left(R=0.94-0.99\right)$ and coefficients of determination $\left({R}^{2}=0.78-0.99\right)$, with $P<0.0001$ in all cases.

To quantify PEF effects on the cytosolic Ca^2+^, pixel intensity was averaged in a ROI drawn manually over the cell body. Fluorescence intensity (F) was background-subtracted and expressed as percent change relative to baseline (∆F = 100% × (F—F_0_) / F_0_), where F_0_ is the mean fluorescence measured in images taken before PEF.

All data were processed in Excel and plotted in Grapher v. 16 (Golden Software, Golden, CO). Grapher was also used to calculate Gaussian and linear regression, correlation coefficients, and fit significance, and to apply a locally weighted scatterplot smoothing (LOWESS) algorithm. Student’s *t*-test was employed to analyze the significance of differences. Error bars are the standard error of the mean, with the number of independent experiments indicated in figure captions. We avoided using special symbols to mark statistical significance in the graphs to preserve their clarity. Instead, the significance level is mentioned in text or figure captions, and can be estimated from the gap between the error bars of the compared groups: A gap exceeding the length of the error bars indicates a significant difference at p < 0.05 (Cumming et al. 2007). For figures showing qualitative results, representative cell images were selected from at least five independent experiments that showed the same effect, and often from 10–20 or more experiments.

## Results

### A Punctate Pattern of PS Externalization Following PEF Exposure

**Fig. 1:**
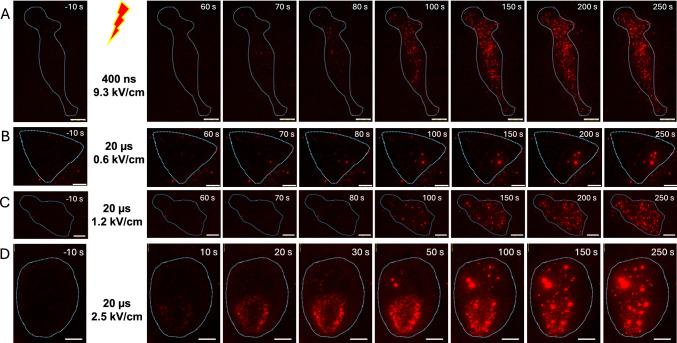
Punctate pattern of PEF-triggered PS externalization. (**A-D**) TIRF images of Annexin V-Alexa Fluor 568 fluorescence in four representative cells, exposed to a single pulse of indicated duration (ns or µs) and intensity (kV/cm) at zero timepoint. The images were taken at indicated times before PEF exposure (left panels) and after it. Blue dotted line contours mark cell outline as seen in bright field images taken before PEF. Scale bar is 10 µm (**A**) or 5 µm (**B-D**). LUT settings are fixed in each time series. Note the different timing of images presented in the bottom row (illustrates the early onset of PS externalization) and the greater diversity of the apparent size and brightness of individual puncta after 20-µs PEFs treatments compared to the 400-ns one

Previous studies reported PEF-induced PS externalization as a relatively slow (seconds to minutes) and diffuse process, primarily at the anode-facing side of the cell. We employed TIRF microscopy with a high-resolution 1.5 NA objective to identify PS externalization associated with individual electropores. TIRF images of Annexin-V-Alexa Fluor 568 fluorescence were acquired at 10 s intervals, starting 60 s before delivery of a single 400-ns or 20-µs pulse and continuing for 5 min minutes afterwards. Contrasting diffuse PS externalization reported previously, we observed multiple, well-defined dots of PS externalization, termed “freckles” (Fig. 1). With 400-ns pulses at or above 9 kV/cm, freckles typically developed with a ~ 1-min delay, and increased in number but not in size over the next several minutes (Fig. 1A). Longer 20-µs pulses at 0.6–2.5 kV/cm produced similar punctate fluorescence patterns (Fig. 1B-D). Brightness and the apparent size of individual freckles within a cell seemed to vary more than after 400-ns PEFs, and stronger pulses induced first freckles as early as in 10 s (Fig. 1D). Still more intense 4.4 kV/cm, 20-µs pulses (data not shown) rapidly produced too many freckles, making it difficult or impossible to isolate individual ones for further analysis.

### Time Course and PEF Dependence of Punctate PS Externalization

**Fig. 2:**
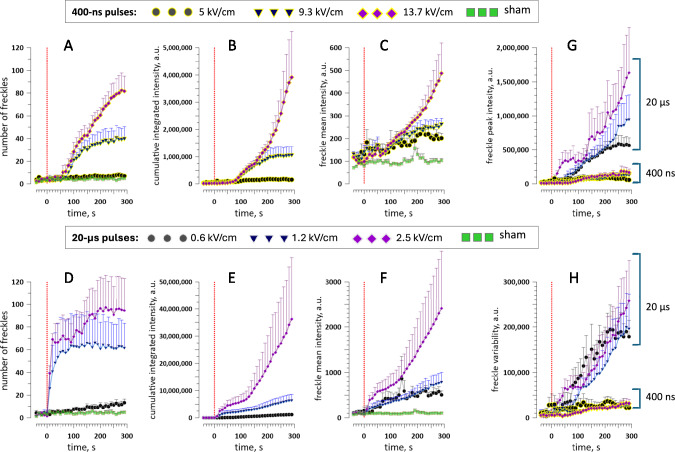
Freckle emergence time course, properties, and dependence on PEF parameters. All measurements were performed within a 10 × 10 µm square ROI, see Methods. In all panels, abscissa is time relative to PEF exposure at 0 s (red dotted line). (**A-C**) Effect of a 400-ns pulse at the indicated intensity (kV/cm) on the number of detected freckles (**A**), the cumulative intensity of all pixels within the freckles (**B**), and the mean intensity of pixels within a freckle (**C**). Sham exposure data are omitted in some panels for visual clarity. (**D-F**) Corresponding plots for 20-µs PEF exposures. Note that y-axes in **E** and **F** span 10 × and 5 × larger ranges than in **B** and **C**, respectively. (**G**, **H**) The peak intensity and variability of freckles induced by 20-µs and 400-ns PEFs combined in the same plots, to highlight the difference in effects of these two PEF types. Mean values ± s.e., n = 4—6. See text for more details

Quantitative analysis of the freckle emerging process was performed as described above in Methods. The automated detection of freckles produced occasional errors; however, in random manual checks, the automated and manual identifications were > 90% consistent. Therefore, the plots in Fig. 2 were generated directly from the automated measurements without further correction.

At 5 kV/cm, 400-ns pulses produced fewer than 10 freckles per a 100-µm^2^ ROI, which was not different from “spontaneous” freckles in sham-exposed cells (which could also be just cell debris or automated detection errors, Fig. 2A). At the same time, the mean intensity was higher than in the control group (Fig. 2C), suggesting that 5 kV/cm was at the threshold for freckle induction. More intense 400-ns PEFs, at 9.3 and 13.7 kV/cm, induced freckles with a ~ 1-min lag, after which their number gradually increased respectively to ~ 40 and ~ 80 per ROI (Fig. 2A). Cumulative integrated intensity (Fig. 2B) stabilized within 3 min of exposure at 9.3 kV/cm but kept climbing throughout the observation period after 13.7 kV/cm. The mean intensity of freckles showed a similar time course (Fig. 2C).

While 20-µs pulses at 0.6, 1.2, and 2.5 kV/cm induced numbers of freckles comparable to 400-ns pulses (Fig. 2D), there was no lag time, and freckles typically were observed in the first frame at 10 s after the pulse. Despite emerging in similar numbers as after 400-ns PEFs, these freckles exhibited as much as tenfold larger cumulative integrated intensity and ~ fivefold larger mean intensity (Fig. 2E and F; note Y-scales different from B and C). Striking differences in freckle peak intensity and variability were highlighted by plotting data for 400-ns and 20-µs PEFs together in Fig. 2G and H.

These results show that PEF effects at 400 ns and 20 µs are substantially different and cannot be adequately described by any single criterion. For example, 20-µs PEF at 0.6 kV/cm produced far fewer freckles than 400-ns PEFs at 9.3 and 13.7 kV/cm (13.3 ± 3 vs 40.5 ± 10 and 81.4 ± 13 freckles per ROI by the end of recording, respectively; p < 0.01 between any of the groups). However, these fewer freckles were on average much brighter, as evidenced by 5- to tenfold differences in both mean and peak intensities (Fig. 2C, F, and G). Concurrently, the variability across individual freckles in a cell (Fig. 2H) was apparently independent of the pulse strength but strongly dependent on pulse duration (tenfold larger for longer 20-µs PEFs, p < 0.01 between any groups of different PEF duration).

To summarize, 400-ns PEFs produced relatively dim and uniform freckles that also appeared after a ~ 1-min delay; freckles after 20-µs pulses appeared with < 10 s delay, were highly variable and, on average, were significantly brighter.

### Lactadherin vs Annexin Detection of PS Externalization

Two principal hypothesized mechanisms of PEF-induced PS externalization are Ca^2+^-independent lateral drift through electropores and activation of scramblases by Ca^2+^ influx. Hence, testing the role of Ca^2+^ in freckle formation could isolate one of these mechanisms. However, Annexin binding to PS is strongly Ca^2+^ dependent, making it unsuitable for PS detection in such experiments. Instead, we used an established Ca^2+^-independent PS tag, FITC-conjugated lactadherin (Shi et al. 2006; Waehrens et al. 2009). As a first step, we tested both dyes together in the physiological solution with Ca^2+^, to adjust lactadherin concentration and imaging protocols to achieve freckle detection comparable to annexin.

**Fig. 3:**
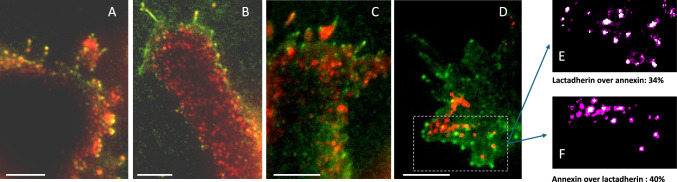
Differential labeling of PEF-induced PS externalization by Annexin V-Alexa Fluor 568 (red) and lactadherin-FITC (green). PEF exposures were performed in the presence of both dyes, and shown are representative frames from a time lapse series of images. (**A**) 130 s after a 2-µs, 5 kV/cm pulse. (**B**) 300 s after a 400-ns, 9.3 kV/cm pulse. (**C**, **D**) two membrane areas of another cell 160 s after a 400-ns, 9.3 kV/cm pulse. (**E**, **F**) Object-based colocalization analysis of the boxed region in **D**. In (**E**), lactadherin-positive objects are shown in pink and pixels within these objects that are also annexin-positive are shown in white. (**F**) shows the reciprocal analysis, with annexin-positive objects as reference. Percent overlap is reported in legends relative to the reference channel. Bars are 5 µm

While lactadherin revealed a similar dotted pattern of PS externalization after PEF, the dots (freckles) exhibited just partial colocalization with those labeled with annexin (Fig. 3). Lactadherin labeling often appeared closer to cell periphery (Fig. 3A and B); this could be related to its larger molecular size and less efficient penetration between cell bottom and the coverslip. However, this factor did not fully explain differences in labeling, such as shown in Fig. 3C and D, with interspersed green, red, and yellow freckles (positive for lactadherin, annexin, or both, respectively). Colocalization measured with MetaMorph typically fell between 30 and 60%, varying between cells and different regions in the same cells, and depending on the object parameters selected in the software (e.g., Fig. 3E and F). Just partial colocalization of PS staining by annexin and by lactadherin was noted in other studies and, at least in some cases, could be related to membrane curvature (Carman et al. 2023).

### Removal of Ca^2+^ Inhibits Freckle Formation

**Fig. 4:**
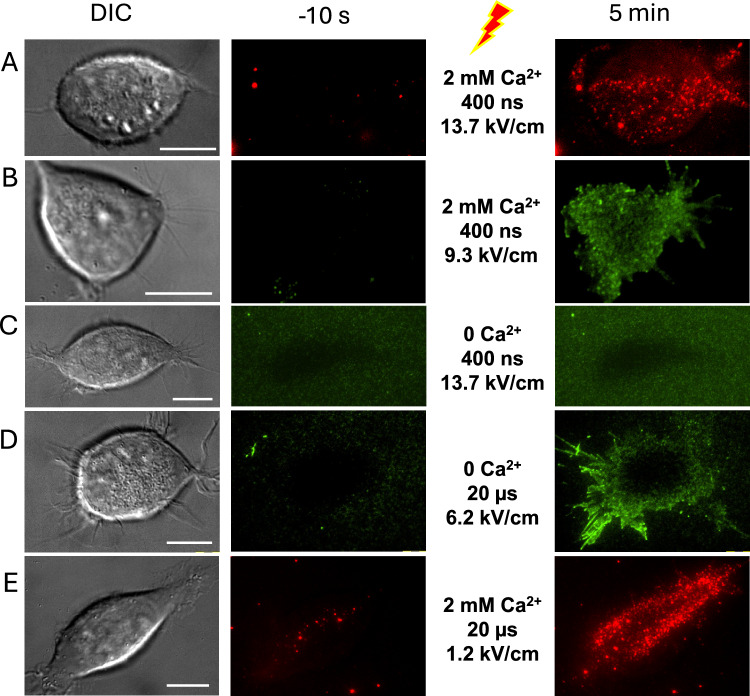
Removal of the extracellular Ca^2+^ inhibits freckle induction by PEFs. (**A**–**C**) Different cells treated as indicated in the legends. Left column, DIC images of the cells prior to PEF exposure. Middle and right columns, respectively, show images of Annexin V-Alexa Fluor 568 (red) or lactadherin-FITC (green) fluorescence prior to PEF delivery and 5 min after it. (**A**, **B**) In the presence of 2 mM extracellular Ca^2^⁺, both dyes labeled freckles similarly. (**C**) Even at 13.7 kV/cm, 400-ns PEF failed to induce freckles. (**D**) A 20-µs PEF at a high electric field strength of 6.2 kV/cm induced freckles even in the absence of extracellular Ca^2+^. (**E**) Freckle induction was more efficient in the presence of 2 mM extracellular Ca^2^⁺ despite application of a fivefold weaker 20-µs pulse. Bars are 10 µm

The effect of extracellular Ca^2+^ on freckle formation is illustrated in Fig. 4. With 2 mM Ca^2+^ present in the medium, cells exposed to 400-ns pulses at 9.3 – 13.7 kV/cm exhibited multiple PS-positive dots when labeled with either annexin or lactadherin (Fig. 4A and B). Exposures in the Ca^2+−^free buffer with 2 mM EGTA produced no freckles in any of 14 experiments where cells were exposed to 400-ns pulses at 9.3—16.8 kV/cm. We could only observe a darker contour of cells due to the exclusion of the dye weakly fluorescent in the extracellular buffer (Fig. 4C). However, 20-µs PEFs were still able to induce freckles, although only at rather high pulse intensities (in 3 out of 3 cells at 6.2 kV/cm and in 6 out of 12 cells at 4.4 kV/cm; Fig. 4D). For comparison, pulsing at 1.2 kV/cm with 2 mM Ca^2+^ produced PS externalization comparable to or stronger than 6.2 kV/cm in the absence of Ca^2+^ (Fig. 4D and E).

In summary, the experiments proved critical role of Ca^2+^ in freckle formation by PEF. The fact that intense PEFs (> 8 times stronger than already effective 20-µs, 0.6 kV/cm PEFs, Fig. 2D-H) triggered PS externalization without extracellular Ca^2+^ could be related to PEF-induced Ca^2+^ mobilization from intracellular stores (Hanna et al. 2017).

### Freckle Mobility

**Fig. 5:**
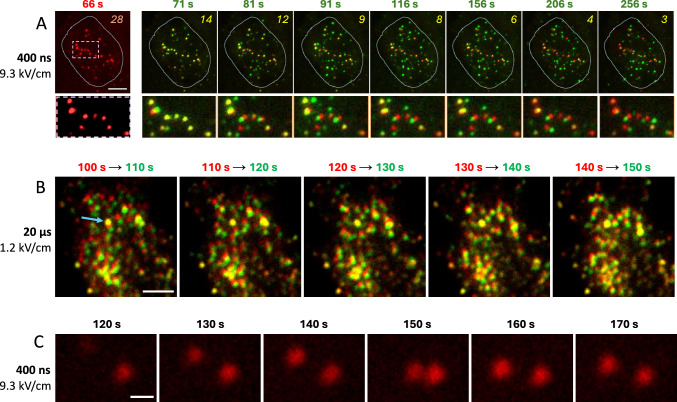
Freckles display stochastic lateral mobility. Images show Annexin V-Alexa Fluor 568 fluorescence at indicated times after a 400-ns, 9.3 kV/cm pulse (**A**, **C**) and after a 20-µs, 1.2 kV/cm pulse (**B**). (**A**) Fluorescence signal at 66 s after PEF was taken as a reference and assigned red color. Subsequent images were assigned green color, made semi-transparent, and laid over the reference image. In these composite images, freckles that stayed at the original location appear yellow; those which moved or emerged anew appear green; and those in red are from the reference image. Numbers at the top right are the original count of freckles (left image) and the number of freckles staying at the original location (i.e., appearing yellow). A dotted line contour is the outline of the cell footprint. A white dashed line rectangle (left panel) identifies the area shown magnified underneath. Bar: 5 µm. (**B**) Images were assembled similarly to (**A**), but instead of a single reference image, there was a constant 10-s interval between the red (preceding) and green (new) images. An arrow in the left panel points to one spot that remained immobile (yellow) despite being surrounded by many mobile ones. Bar: 2 µm. (**C**) An example of two freckles traveling toward each other and then spreading apart after a “kiss”. Bar: 0.5 µm. See text for more details

Figure 5 illustrates the apparently stochastic movement of freckles over time. In panel A, freckles observed at 66 s after a 400-ns, 9.3 kV/cm pulse were taken as a reference and assigned red color. Subsequent recordings, at indicated times, were assigned green color, made semi-transparent, and overlaid on the reference image. In these composite images, freckles that remained at the same location as in the reference image appeared yellow; those which moved at least once during the observation period or emerged anew appeared green; and those in red were from the reference image (unless they were overlapped by another freckle in a later recording, which would make them yellow). Out of 28 freckles counted in the reference image, 14 (50%) shifted already in 5 s and 14 did not; and 3 out of the latter group remained immobile even 3 min later.

Figure 5B (100 to 150 s after a 20-µs, 1.2 kV/cm pulse) is built similarly but, instead of a single reference image, there was a constant 10-s interval between the red (preceding) and green (new) images. An arrow in the left panel points to a single freckle that remained immobile (yellow) throughout the experiment, no matter that it was surrounded from all directions by freckles that moved continually, changing their location relative to each reference frame. Such moving behavior, which was typical for all tested PEF exposure parameters, is difficult to explain if freckles are indeed patches of externalized PS on electroporated cell membrane. Both the mobile and the immobile freckles emerged after PEF exposure, indicating that neither was attributable to occasional fluorescent debris.

**Fig. 6:**
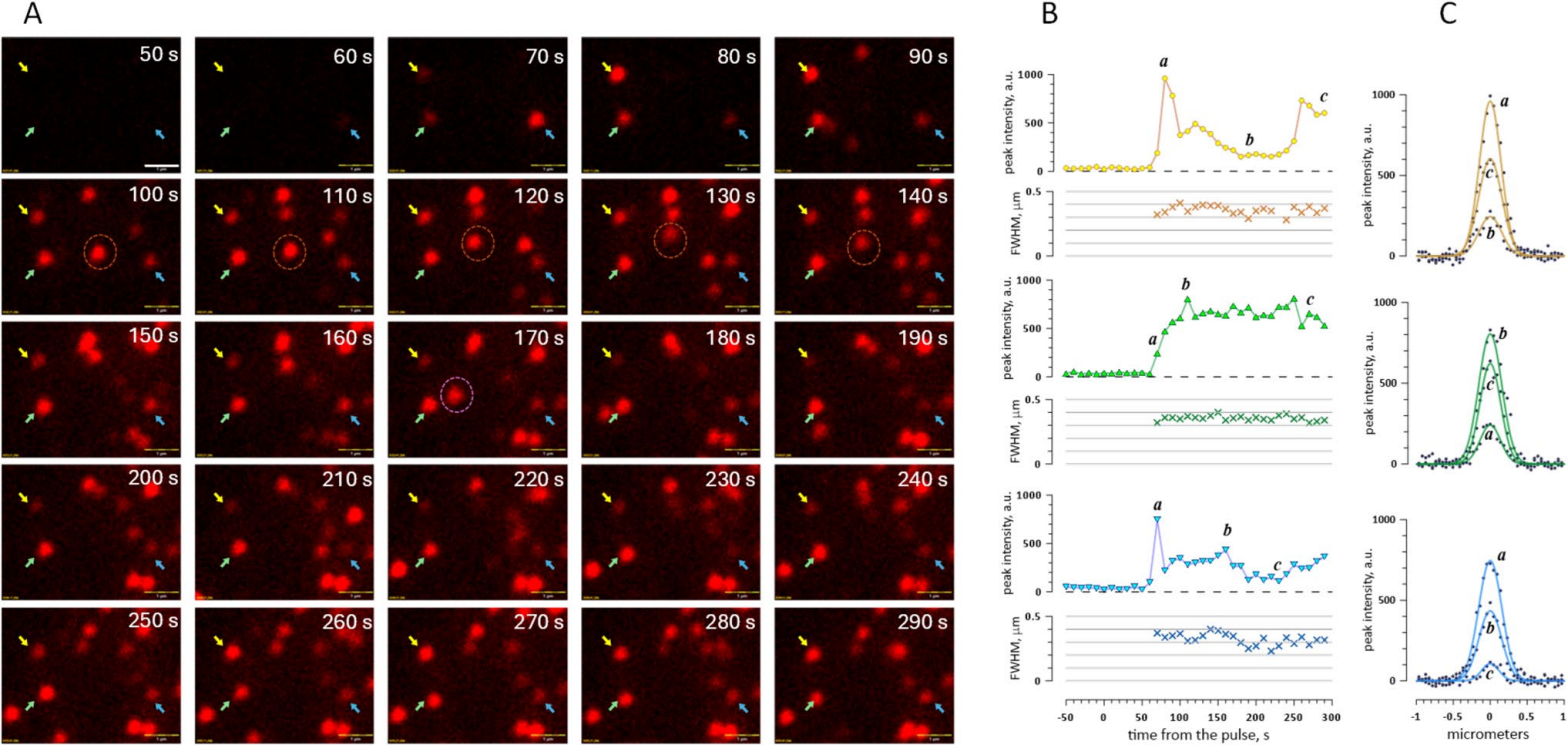
Emergence and time evolution of freckles. (**A**) Time lapse TIRF images of Annexin V-Alexa Fluor 568 fluorescence at the indicated times after a 400-ns, 9.3 kV/cm pulse applied at 0 s. Three colored arrows mark freckles that appeared first. The arrows are stationary across all images and serve as positional landmarks. Freckles outlined by dotted line circles at 100 – 140 s and at 170 s are examples of their abrupt appearance and disappearance. (**B**) Time courses of the peak intensity and apparent size of the three freckles marked by the colored arrows. The yellow, green, and blue traces correspond to the freckles indicated by the yellow, green, and blue arrows, respectively. The plotted values were derived from Gaussian fits to line scans through the freckles at each timepoint. Line scans examples and fits are illustrated in (**C**) for the representative timepoints **a**, **b**, and **c**. Freckle size was expressed as the full width at half maximum (FWHM) of the fitted profiles. See text for details

Figures 5C and 6A show more examples of freckle motion that appear unlikely for PS patches on the membrane. In Fig. 5C (400 ns at 9.3 kV/cm), two freckles come together for a “kiss” and spread apart with no appreciable changes in their brightness. Such motion would require the membrane between the freckles to fold sharply while not pulling the freckles themselves away from the glass substrate (with evanescence field penetration depth of only ~ 100 nm above the coverslip, even a subtle vertical movement of freckles should change their brightness).

In Fig. 6A, colored arrows point to three freckles that appeared early after a 400-ns, 9.3 kV/cm exposure. The arrows are stationary across all images and serve as position landmarks. The three freckles showed only minor lateral movement over the course of the experiment while their brightness fluctuated a lot and asynchronously. For example, the freckle at the top (yellow arrow) peaked in brightness at 80 s, while one at the bottom (blue arrow) dimmed down from its peak at 70 s. To quantify brightness changes over time, a 50-pixel long and 2-pixel wide ROI was placed across the center of each freckle; if the freckle moved, even slightly, the line position was adjusted accordingly. Pixel intensity measured along each ROI closely followed a Gaussian distribution (with correlation coefficients > 0.9), and the maximum of the Gaussian fits was plotted as peak intensity of the freckles in time (Fig. 6B and C). In one of the freckles (green arrow), the peak intensity gradually increased and then remained nearly stable, which could be explained simply by binding more fluorescently-tagged annexin with time. However, this explanation is not suitable for two other freckles, which peaked in the beginning and then became 4–5-fold dimmer, with partial recovery after the dip. Such behavior could only be attributed to stochastic vertical movements, which happened in an asynchronous and seemingly independent fashion despite the close proximity of the freckles to each other. Likewise, the vertical shift was the most likely cause of abrupt appearance and disappearance of two other freckles (marked by dotted line circles, at 100 – 140 s and at 170 s).

The stochastic mobility of freckles, both vertically and horizontally, and often independently from each other, was the first indication that they are not necessarily connected to the cell membrane. Below we provide more evidence that what was expected to be PS patches were in fact PS-positive vesicles shed by electroporated cells.

### Freckle Size Measured From Gaussian Fits is Nearly Constant and Independent of Brightness

Trying to figure out freckle size directly from their images is deceptive, because their apparent size depends on visual brightness and the look-up table (LUT) settings, and there is no definitive freckle border but a gradual brightness decline. The fact that pixel intensity across a freckle closely follows Gaussian distribution offers a more reliable method of defining their size through the full width at half maximum (FWHM) of Gaussian fits. Fig. 6B shows that FWHM was essentially independent from the pixel intensity and nearly the same (0.3–0.4 µm) for the three freckles. This observation is consistent with freckles being fluorescent vesicles whose vertical movement in the evanescence field changes their brightness but not diameter.

**Fig. 7:**
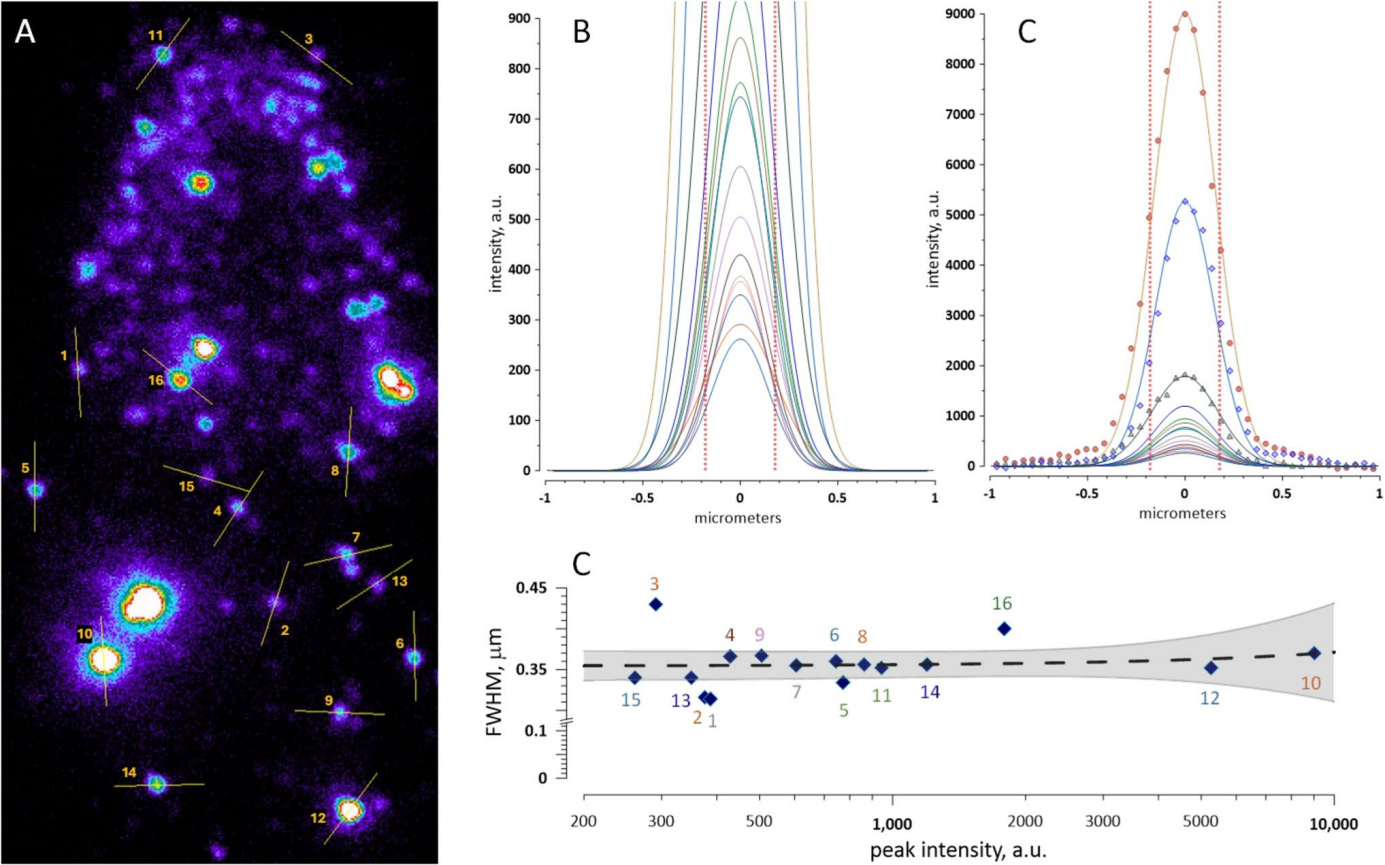
Freckles exhibit a Gaussian intensity profile with constant FWHM independent of intensity. **(A)** TIRF image of freckles labeled with Annexin V-Alexa Fluor 568 in a representative cell, 2 min after a 20-µs, 2.4 kV/cm pulse. Pixel intensity is shown in pseudocolor. Numbered line ROIs were drawn across 16 individual freckles. All ROIs were 50 pixels long and 2 pixels wide (2.3 × 0.92 µm). The lines were positioned at various angles to avoid or minimize signal from neighboring freckles. **(B, C)** Gaussian fits to the line scans through the selected freckles, plotted on vertical scales differing by a factor of 10. Vertical dotted lines indicate the FWHM, which was nearly identical for all fits. For clarity, the actual line-scan data are not shown except for three freckles in **(C)**. **(D)** FWHM of freckles 1–16 plotted against their peak intensity. Freckle numbers match those in image **(A)**, and font colors match the Gaussian profile colors in **(B)** and **(C)**. The dashed line is the linear fit to the data, and the shaded area is the 95% confidence interval of the fit

**Fig. 8:**
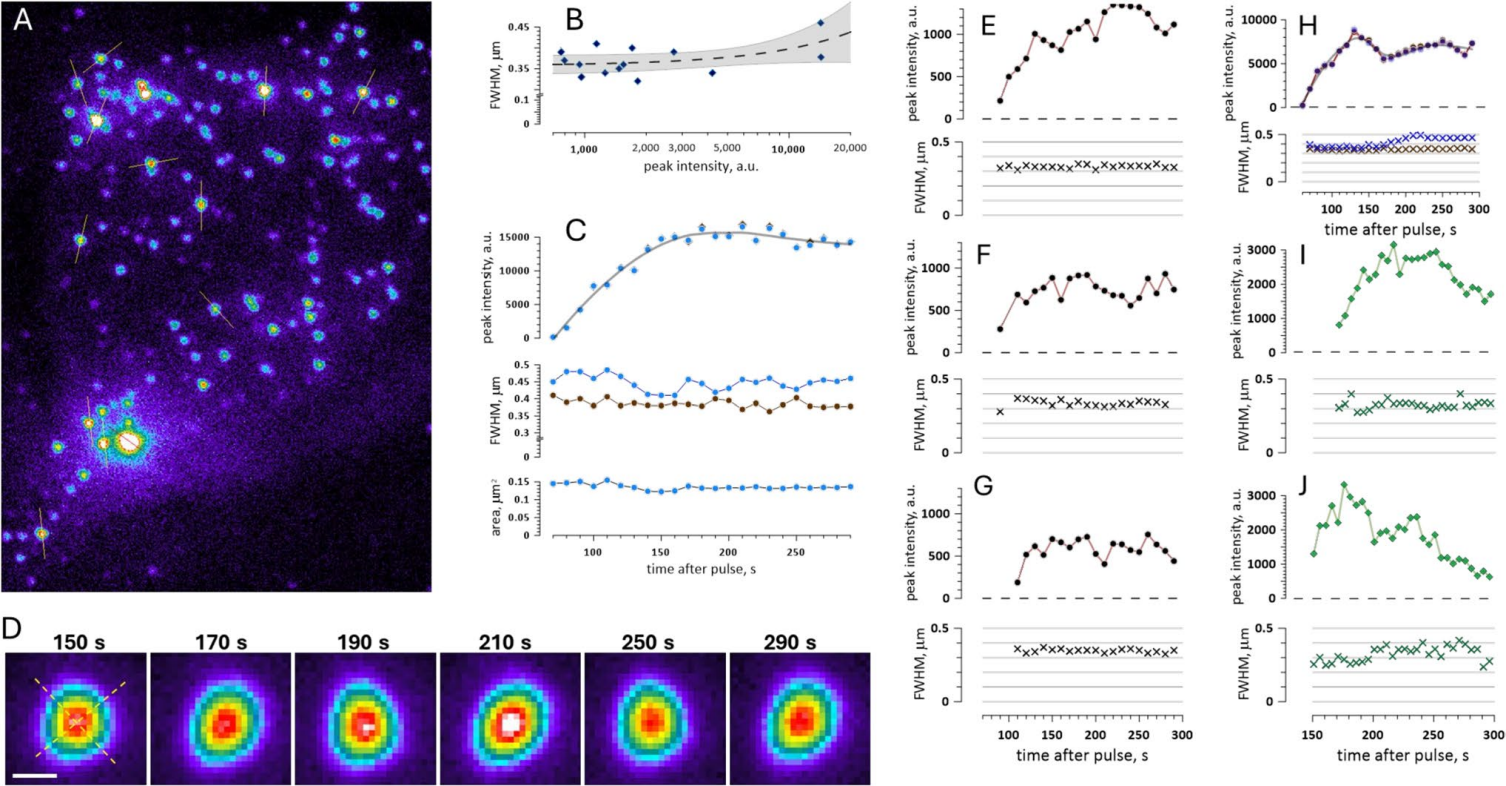
Freckle profiles and evolution across diverse experimental conditions. **(A)** TIRF image of freckles labeled with Annexin V-Alexa Fluor 568 in a representative cell, 5 min after a 400-ns, 9.3 kV/cm pulse. Pixel intensity is shown in pseudocolor, and 50-pixel long, 2-pixel wide line ROIs were drawn across selected freckles. **(B)** These line scans were fitted with Gaussians (same as in Fig. 7B and C; not shown here), and FWHM of the fits was plotted against their peak amplitude. The dashed line and the shaded area are the linear fit to the data and its 95% confidence interval. **(C)** The time course of the peak intensity, FWHM, and apparent area of the largest freckle from (**A**). FWHM was measured from two orthogonal line scans, as shown in the left image in **(D)**. The area was calculated assuming freckle as an oval with the diameters equal to the orthogonal FWHM measurements. **(D)** The same freckle at a larger magnification at indicated times after PEF. Bar: 0.5 µm. **(E-J)** Examples of time evolution of freckle intensity and FWHM in different cells, labeled with either Annexin V-Alexa Fluor 568 **(E–H)** or FITC-lactadherin **(I, J).** The cells were exposed to 400-ns, 9.3 kV/cm pulses except 20-µs, 0.6 kV/cm for **(H)**, which represents an unusually large freckle that was measured in two orthogonal directions (brown and blue symbols)

The lack of correlation of FWHM with brightness is explored further in Figs. 7 and 8. Fig. 7A shows Annexin-labeled freckles at 2 min after a 20-µs, 2.5 kV/cm pulse. Different brightness of individual freckles is emphasized by the pseudocolor; the LUT was chosen for the best visualization of both dim and bright freckles, resulting in visual saturation of the brightest spots (the actual pixel values were far below saturation). Linear ROIs (50-pixel long and 2-pixel wide; one pixel is 46 nm) were placed over randomly selected freckles at angles chosen to avoid or minimize readings from the nearby freckles. Fig. 7B and C shows Gaussian fits through these 16 ROIs, at tenfold different scales. For clarity, the actual measured pixel values are shown only for several selected ROIs. Fig. 7D presents measured FWHM as a function of the peak pixel intensity, which ranged as much as 40-fold across the selected freckles. Despite this profound difference in brightness, FWHM was nearly the same for all the freckles, centering at ~ 0.35 µm.

Figure 8A and B provides a similar analysis in a cell image taken 5 min after a 400-ns, 9.3 kV/cm pulse. Despite the difference in pulse parameters and timing, FWHM in most freckles centered again at about 0.35 µm. For the brightest spot in Fig. 8A, line scans were performed in time lapse images and in two orthogonal directions (Fig. 8C and D). The peak intensity through the two perpendicular scans expectedly produced nearly the same numbers (Fig. 8C, top); it steadily increased from 150 a.u. at 80 s when the freckle was first detected to ~ 15,000 a.u. 70–100 s later, and then remained nearly stable. In spite of this 100-fold brightness change, FWHM displayed only small fluctuations, between 0.38 and 0.48 µm. Remarkably, between 150 and 290 s FWHM fluctuations in two orthogonal directions mirrored each other, i.e., elongation in one direction was accompanied by shortening in the perpendicular direction. These fluctuations between round and oval shapes are also noticeable by eye in high-magnification images of this freckle in Fig. 8D.

Figure 8E and K presents more examples of the time evolution of freckles in different experiments, including when they were labeled with lactadherin (panels I and J). For the most part, peak intensity increased in the beginning, followed by a random fluctuation, plateauing, or decrease (could be caused by bleaching). However, FWHM only varied between 0.3 and 0.4 µm (up to 0.5 µm in a single case), with minor random scatter attributable to fitting variability rather than any underlying trend.

If freckles represented PS patches within the plasma membrane, lateral diffusion of PS would be expected to produce substantial spot broadening during the experiment. With reported PS diffusion coefficients of ~ 0.2–1 μm^2^/s (Kay et al. 2012), these patches should disperse over micrometer-scale distances within minutes. The essentially constant FWHM of freckles thus provides strong additional evidence that they are vesicles rather than PS patches.

### Deriving Freckle Size From FWHM

Gaussian line-scan profiles that remained stable over time and across diverse experimental conditions, with FWHM values close to the diffraction limit, suggested that the apparent freckle width was governed primarily by the point spread function (PSF). However, recordings that showed changes in freckle shape and reciprocal fluctuations of FWHM along orthogonal axes, as in Fig. 8C and D, indicated that the observed footprint reflected not only PSF blurring but also finite object size. To estimate freckle diameter, we treated freckles as spherical objects and determined the PSF experimentally using size-calibrated beads (Cole et al. 2011), rather than relying on an idealized theoretical PSF.

Calibrated 110-nm fluorescent beads were imaged, and pixel intensity was measured using line scans in the same way as for freckles. For 570-nm excitation and 630-nm emission (the same settings as for Annexin V–Alexa Fluor 568), the measured bead width had ${\mathrm{F}\mathrm{W}\mathrm{H}\mathrm{M}}_{\mathrm{b}\mathrm{e}\mathrm{a}\mathrm{d},\mathrm{m}\mathrm{e}\mathrm{a}\mathrm{s}}=308$ nm.

With TIRF penetration depth $d=100$ nm, the excitation intensity falls to half of its interface value at height ${z}_{1/2}$ above the glass surface.

$${z}_{1/2}=d\mathrm{l}\mathrm{n}2=100\mathrm{l}\mathrm{n}2\approx 69\text{ nm}$$

Thus, in a fluorescent sphere of diameter $D$ resting on the glass, emission falls to 50% of its maximum at a contour located 69 nm above the glass. The diameter of this contour, i.e. the intrinsic bead width that would be measured in the absence of PSF blur, is.

$${\mathrm{F}\mathrm{W}\mathrm{H}\mathrm{M}}_{\mathrm{b}\mathrm{e}\mathrm{a}\mathrm{d},\mathrm{i}\mathrm{n}\mathrm{t}}=2\sqrt{{D}_{\mathrm{b}\mathrm{e}\mathrm{a}\mathrm{d}}{z}_{1/2}-{z}_{1/2}^{2}}=2\sqrt{110\cdot 69-{69}^{2}}\approx 106\text{ nm}$$

Assuming approximate quadrature addition of intrinsic object width and PSF blur, the effective lateral PSF width can be estimated as.

$${\mathrm{F}\mathrm{W}\mathrm{H}\mathrm{M}}_{\mathrm{P}\mathrm{S}\mathrm{F}}=\sqrt{{\mathrm{F}\mathrm{W}\mathrm{H}\mathrm{M}}_{\mathrm{b}\mathrm{e}\mathrm{a}\mathrm{d},\mathrm{m}\mathrm{e}\mathrm{a}\mathrm{s}}^{2}-{\mathrm{F}\mathrm{W}\mathrm{H}\mathrm{M}}_{\mathrm{b}\mathrm{e}\mathrm{a}\mathrm{d},\mathrm{i}\mathrm{n}\mathrm{t}}^{2}}=\sqrt{{308}^{2}-{106.2}^{2}}\approx 289\text{ nm}$$

The ideal diffraction-limited FWHM for a point emitter imaged with a 1.50 NA objective is approximately 181 nm at 530-nm emission and 216 nm at 630-nm emission. The bead-derived FWHM was larger, reflecting the effective resolution of our full TIRF imaging and analysis pipeline, including the substrate, filters, camera sampling, and line-scan fitting. We therefore used the experimentally measured effective PSF, rather than the idealized theoretical value, for subsequent deblurring calculations. We then applied the same approximation to deblur the average measured freckle width of 350 nm.

$${\mathrm{F}\mathrm{W}\mathrm{H}\mathrm{M}}_{\mathrm{f}\mathrm{r}\mathrm{e}\mathrm{c}\mathrm{k}\mathrm{l}\mathrm{e},\mathrm{d}\mathrm{e}\mathrm{b}\mathrm{l}\mathrm{u}\mathrm{r}}=\sqrt{{\mathrm{F}\mathrm{W}\mathrm{H}\mathrm{M}}_{\mathrm{f}\mathrm{r}\mathrm{e}\mathrm{c}\mathrm{k}\mathrm{l}\mathrm{e},\mathrm{m}\mathrm{e}\mathrm{a}\mathrm{s}}^{2}-{\mathrm{F}\mathrm{W}\mathrm{H}\mathrm{M}}_{\mathrm{P}\mathrm{S}\mathrm{F}}^{2}}=\sqrt{{350}^{2}-{289}^{2}}\approx 197\text{ nm}$$

Finally, we converted the deblurred footprint into the physical freckle diameter $D$ using the same sphere-TIRF geometry and ${z}_{1/2}=69$ nm.

$$D=\frac{{\left({\mathrm{F}\mathrm{W}\mathrm{H}\mathrm{M}}_{\mathrm{f}\mathrm{r}\mathrm{e}\mathrm{c}\mathrm{k}\mathrm{l}\mathrm{e},\mathrm{d}\mathrm{e}\mathrm{b}\mathrm{l}\mathrm{u}\mathrm{r}}/2\right)}^{2}+{z}_{1/2}^{2}}{{z}_{1/2}}=\frac{(197/2{)}^{2}+{69.3}^{2}}{69.3}\approx 210\text{ nm}$$

These calculations also show that relatively small variations in measured FWHM may correspond to large differences in the physical diameter of freckles. Rare oversized freckles (such as in Fig. 8C, D, and H) had FWHM values up to 400–450 nm, corresponding to physical diameters of approximately 345–500 nm.

The same measurements of 110-nm beads using FITC-lactadherin excitation and emission settings (488-nm excitation, 530-nm emission) yielded a slightly larger ${\mathrm{F}\mathrm{W}\mathrm{H}\mathrm{M}}_{\mathrm{P}\mathrm{S}\mathrm{F}}$ of 312 nm, despite the shorter wavelength that would be expected to reduce PSF width. Accordingly, a measured ${\mathrm{F}\mathrm{W}\mathrm{H}\mathrm{M}}_{\mathrm{f}\mathrm{r}\mathrm{e}\mathrm{c}\mathrm{k}\mathrm{l}\mathrm{e},\mathrm{m}\mathrm{e}\mathrm{a}\mathrm{s}}$ of 350 nm translated into a smaller estimated physical diameter of about 160 nm.

We attribute these modest discrepancies between estimates obtained under different optical settings to methodological uncertainties rather than to actual differences in freckle size. The calculated diameters should therefore be regarded as approximate estimates, derived from PSF deblurring and a simplified sphere-in-TIRF geometry, rather than direct super-resolution measurements. Within these limitations, most freckles are consistent with submicron vesicles of about 200 nm in diameter, whereas rare oversized freckles may reach several hundred nanometers and could represent doublets or overlapping smaller freckles.

### Freckles are Extracellular Vesicles

**Fig. 9:**
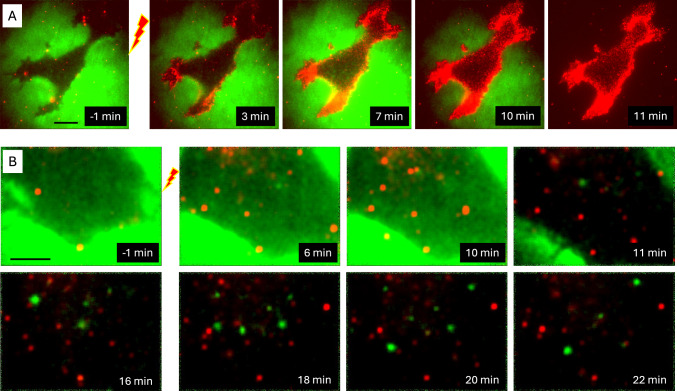
Emerging PS-positive freckles do not take up extracellular dextran. **(A, B)** Time-lapse images from two separate experiments in which cells were exposed to a 400-ns, 6.2 kV/cm pulse in the presence of two dyes, Annexin V–Alexa Fluor 568 and FITC-labeled 10-kDa dextran (red and green, respectively). Labels indicate the time before and after PEF exposure. At ~ 10 min, cells were washed with dye-free physiological solution. All images in **(A)** use the same LUT. In **(B)**, LUTs were adjusted for visual clarity: the first three images share one LUT, the fourth uses a different LUT, and all images in the bottom row share another LUT. Scale bars: 10 µm in **(A)** and 5 µm in **(B)**. Note the lack of detectable dextran-FITC staining after the wash in **(A)** and the lack of colocalization between structures stained with dextran-FITC and Annexin V–Alexa Fluor 568 in **(B)**

Previous experiments did not determine whether freckles formed inside the cell through endocytosis, or outside the cell through ectocytosis or exosome secretion, or it was a combination of these processes. To distinguish among these mechanisms, the extracellular solution was supplemented with both Annexin V–Alexa Fluor 568 and FITC-labeled 10 kDa dextran. Owing to its relatively large size (hydrodynamic radius ~ 2 nm (Armstrong et al. 2004)), dextran showed no detectable entry into electroporated cells. Before PEF exposure, cells appeared as dark silhouettes against a brightly fluorescent extracellular background (Fig. 9), with only a few spontaneous Annexin-positive spots. In the two representative cells shown in Fig. 9A and B, a 400-ns pulse at 6.2 kV/cm induced, respectively, massive and moderate formation of Annexin-positive freckles. The partial overlap of the two fluorescence channels, seen as yellow signal at 7–10 min, was likely due to light scattering and apparent channel bleed-through rather than true colocalization. To validate this interpretation, perfusion with dye-free physiological solution was initiated 9–10 min after PEF. In the subsequent images, extracellular fluorescence was markedly reduced or absent, and no green signal of FITC dextran was detected within the freckles.

Notably, Fig. 9B at 11–22 min shows both Annexin-positive and dextran-positive puncta. Most moved stochastically within the field of view but never colocalized. These observations suggest that Annexin-positive freckles originated from the cell interior without trapping extracellular FITC-dextran and were formed by either exocytosis or ectocytosis. The mechanism responsible for the formation of these PS-positive microvesicles remains to be established. In contrast, the FITC-dextran-positive puncta were likely PS-negative endocytic vesicles; whether this endocytosis was spontaneous or PEF-induced remains unknown.

### Freckles Form at Supra-Electroporative PEF Intensities

Prior interpretations of PS externalization in electroporated cells focused on diffuse PS exposure across the plasma membrane. Shedding extracellular vesicles as a mechanism of PS externalization has not been anticipated or considered. It was therefore important to determine where freckle formation falls on the scale of electric field strengths known to cause electroporation. This comparison does not by itself prove a causal relationship, but it does establish whether freckle formation occurs within the same range of field intensities as membrane permeabilization.

To define the electroporation threshold, we used cytosolic Ca^2+^ elevation as a sensitive and well-established endpoint. HEK cells lack voltage-gated Ca^2+^ channels (Hristov et al. 2018), so cytosolic Ca^2+^ elevation after PEF can occur either through electroporative influx across the plasma membrane or through Ca^2+^ release from intracellular stores. However, intracellular Ca^2+^ release requires higher field intensities than plasma membrane permeabilization (Semenov et al. 2013) and, for the purposes of this analysis, can be neglected.

**Fig. 10:**
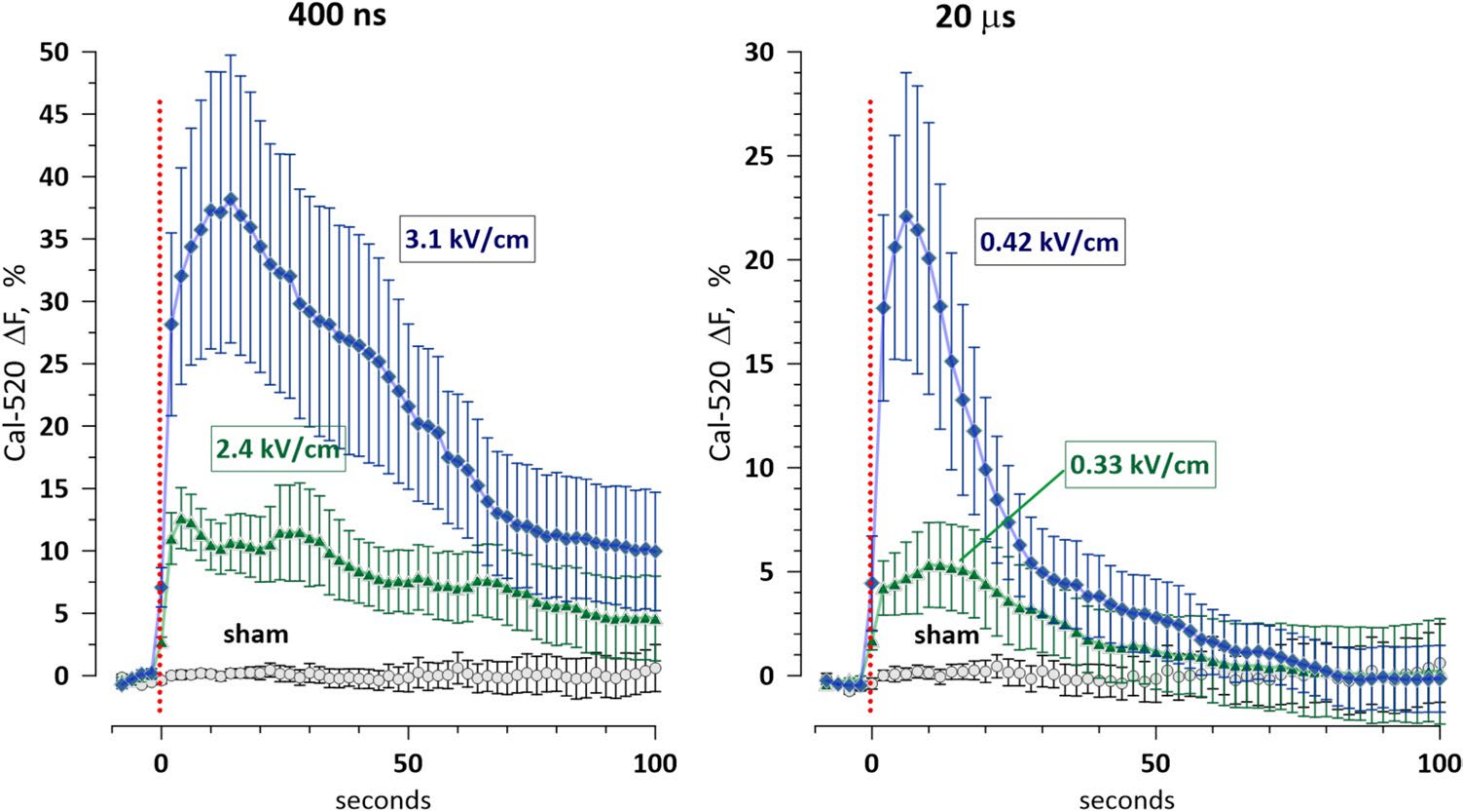
Electroporative uptake of Ca^2+^ is triggered at PEF strengths below those that induce freckles. The graphs show the transient increase in the fluorescence of the Ca^2+^-sensitive intracellular dye Cal-520 following PEF exposure (red dotted line). Pulse widths and strengths are labeled in the graphs. Sham-exposed samples were treated identically but PEF amplitude was set to zero. Mean ± s.e., n = 10–18. Differences between the groups in each graph are significant at p < 0.05 or better (unpaired two-tailed *t* test with Dunnet’s correction)

Figure 10 shows the time course and dose dependence of cytosolic Ca^2+^ elevation induced by 400-ns and 20-µs pulses. Statistically significant responses ($p<0.01$) were observed at 2.4 and 0.33 kV/cm, respectively. These values are approximately twofold lower than the electric field strengths that were near threshold for freckle induction by 400-ns and 20-µs pulses, respectively (Fig. 2). We therefore conclude that freckles form at electric field strengths above the electroporation threshold. This finding supports the interpretation that freckle formation is a downstream consequence of electroporation, whether mediated by Ca^2+^ influx and activation of scramblases or by another mechanism. The exact pathway, however, remains to be determined.

### Opposite Field Polarity is Similarly Effective in Triggering Freckle Formation

PS is charged negatively, and one of discussed mechanisms of its externalization includes the electrophoretic drift through the membrane towards the anode (Hu et al. 2005; Tekle et al. 2008; Vernier et al. 2006a, 2006b). In the experiments described above, however, the ITO surface was negative (cathode) relative to the rod electrode positioned above the cells, which already argued against the electrophoresis-guided mechanism. Nevertheless, the opposite field polarity was tested to determine whether it would increase the efficiency of freckle formation or produce qualitatively different patterns of PS externalization.

This experiment concurrently addressed another potentially relevant mechanism. Recent studies have linked electropermeabilization to oxidation of membrane lipids (Breton and Mir 2018; Kotnik et al. 2019; Rems et al. 2019). In our previous work using TIRF imaging and the same PEF exposure technique as in the present study (Michel et al. 2020), no lipid oxidation was detected when the ITO surface served as the cathode. Using ITO as the anode produced readily detectable lipid oxidation; however, the extent of oxidation did not correlate with membrane permeabilization and it was regarded as a side effect rather than the cause of membrane disruption. Because oxidation may loosen lipid packing and thereby facilitate PS translocation, opposite-polarity pulses provided a way to test the possible contributions of both electrophoretic drift and oxidation-dependent membrane remodeling.

**Fig. 11:**
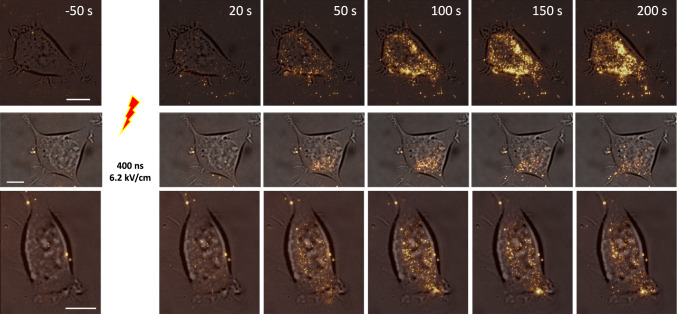
Induction of freckles by “inverted” PEF (ITO surface made positive and the rod electrode above the cell is negative). Time-lapse images of Annexin V–Alexa Fluor 568 fluorescence at indicated times before and after PEF were laid over bright field images taken prior to the experiment. For the best visual contrast, annexin fluorescence is shown here in the gold pseudocolor. Each row is an independent experiment. Bars are 10 µm

As shown in Fig. 11, 400-ns PEFs at 6.2 kV/cm applied in the opposite electric field direction (with ITO made positive and rod negative) triggered PS externalization that was qualitatively similar to that observed with the original polarity. These experiments revealed no obvious increase in efficiency and no distinct pattern of PS externalization dependent on the electric field direction. Thus, in first approximation, the data do not support either electrophoretic drift of PS toward the anode or a major contribution of polarity-dependent lipid oxidation to freckle formation.

Separately, the top-row images in Fig. 11 show clear accumulation of freckles beyond the cell border after PEF exposure. This accumulation was routinely observed in many experiments, regardless of PEF parameters or polarity. Although their exact origin cannot be traced because they may have emerged outside the TIRF excitation zone and entered it later, the presence of freckles beyond the cell border supports their interpretation as extracellular vesicles.

## Discussion

Our study is, to our knowledge, the first to apply TIRF microscopy to analyze PS externalization by electroporating PEFs. Contrary to numerous previous reports of diffuse PS externalization triggered by PEFs, we observed predominantly, if not exclusively, a punctate pattern. These puncta were further identified as extracellular, PS-positive vesicles of approximately 200 nm in diameter. This small size, close to the diffraction limit, was one likely reason why these puncta have not been resolved in earlier studies that utilized conventional fluorescence imaging. Another reason is that electroporation of cells on the microscope stage is commonly performed with electrodes placed on opposite sides of a cell (Bhattacharya et al. 2022; Pakhomov et al. 2007; Vernier et al. 2006a, 2004a), such that permeabilization occurs mainly at the lateral membrane surfaces. Under those conditions, even high-resolution imaging would merge multiple adjacent events into a diffuse signal. In contrast, the present experimental configuration allowed selective imaging of the basal membrane with minimal background, thereby revealing PS externalization as discrete individual events.

Although diffuse PS staining was often present alongside a massive expression of PS puncta, it could represent the scattered or collective signal from multiple unresolved or overlapping freckles. Thus, our results do not establish whether truly diffuse PS externalization occurred at all, at least under the tested PEF conditions and in the present cell model. This result challenges the conventional view of post-electroporation PS externalization as a diffuse event.

Our data also do not support the hypothesis that PS reaches the outer leaflet via lateral drift through electropores (Hu et al. 2005; Vernier et al. 2006a, 2004a, 2004b, 2006b). The lateral drift is expected to produce local, radially expanding patches of PS, but not PS-positive vesicles that also keep emerging for minutes, long time after most electropores reseal (Silkunas et al. 2024, 2023). Pulse polarity did not qualitatively affect freckle formation, arguing against a dominant role of the electrophoretic PS movement across the membrane (Hu et al. 2005). The difference from the previous studies (Hu et al. 2005; Vernier et al. 2006a, 2004a, 2004b, 2006b) could be related to applying multiple, shorter PEFs at higher field strengths (4- to 30-ns pulses at 25–80 kV/cm), versus a single 400-ns or 20-µs pulse at up to 16.8 kV/cm and 6.2 kV/cm, respectively, in the present work. PS externalization was not mediated by membrane blebbing and therefore did not support the proposed mechanism of PS translocation through regions of constrained lipid packing at the base and apex of blebs (Tekle et al. 2008). Finally, lipid oxidation of the plasma membrane adjacent to the ITO electrode was strongly pulse-polarity dependent (Michel et al. 2020), whereas freckle formation was not (Fig. 11); therefore, lipid oxidation was unlikely to mediate PS externalization and vesicle shedding.

In contrast, the strong dependence of vesiculation on extracellular Ca^2+^ (Fig. 3), together with the apparently higher threshold for vesiculation than for electroporation (Figs. 2 and 10), was consistent with electroporative Ca^2+^ entry and subsequent activation of Ca^2+^-dependent scramblases such as TMEM16F (Muratori et al. 2017; Nagata et al. 2020; Wu et al. 2020). Such a mechanism would imply that focal PS externalization in the plasma membrane leads to membrane bulging followed by vesicle scission, although this remains to be demonstrated. Scramblases could also be entrapped within freckles and remain active after their scission from the plasma membrane.

Freckle brightness varied markedly, especially between 400-ns and 20-µs exposures. In TIRF imaging, fluorescence intensity cannot be interpreted solely as the amount of PS per vesicle, because it also depends on vesicle position within the evanescent field, probe accessibility, vesicle size, membrane curvature, and possible overlap of nearby vesicles. The several-fold brightness fluctuations of individual freckles with nearly constant FWHM suggest that axial motion contributed to intensity changes. Thus, brighter freckles after 20-µs pulses may reflect differences in vesicle formation, labeling, or positioning, but their mechanistic basis remains unresolved. We therefore describe these differences as an experimental observation but avoid assigning it a specific functional significance.

Freckle formation may represent a membrane repair response rather than a purely pathological byproduct. Vesicle shedding is a well-established mechanism for removing damaged plasma membrane and restoring membrane integrity (Cooper and McNeil 2015; Demonbreun and McNally 2016; Jimenez and Perez 2017; Scheffer et al. 2014). Hence, freckles may reflect local excision and disposal of membrane regions damaged by electroporation. From this perspective, PEF exposure may provide a useful tool for probing membrane repair with high spatial and temporal precision (Bhattacharya et al. 2022).

Alternatively, freckles could represent exosomes released from multivesicular bodies (MVBs) in response to elevated cytosolic Ca^2+^. HEK293 cells possess a functional MVB pathway and are known to release exosome-type vesicles (Back et al. 2017; Williams et al. 2023). Exosomes can be released by Annexin A6-mediated, Ca^2+^-dependent repair of plasma membrane damage (Williams et al. 2023). Exosomes commonly constitutively expose PS on their outer surface, which readily explains the PS positivity of freckles (Flaskamp et al. 2025). However, the profound differences in brightness and population heterogeneity between freckles induced by 400-ns and 20-µs PEFs (Fig. 2) does not appear consistent with the exosome release mechanism. Instead, they may be related to distinct Ca^2+^ dynamics after “short” and “long” PEFs due to differences in electropore size and formation patterns (Semenov et al. 2013, 2015).

Extracellular vesicles are recognized mediators of intercellular communication, but only a handful of studies have examined their induction by PEFs or their potential role in PEF-based therapies. A study in two melanoma cell lines reported that shedding microvesicles may explain the bystander effect of mediating the death signals to neighboring cells (Prevc et al. 2016). The bystander effect was independently explored in F98 glioma and LL/2 Lewis lung carcinoma cell lines subjected to high-frequency irreversible electroporation (H-FIRE). Supernatants of H-FIRE-treated tumor cells, containing small tumor-derived extracellular vesicles (sTDEV) disrupted endothelial cell monolayer integrity in the Transwell blood–brain barrier model (Murphy et al. 2024). This effect could be related to the altered protein composition of the vesicles suggesting a potential role in modulating post-ablation immune responses and tumor microenvironment (Murphy et al. 2025). In liver cancer cells, nsPEF promoted the release of programmed death-ligand 1 (PD-L1), a key immune checkpoint molecule, via extracellular vesicles of different sizes, leading to the dysfunction of CD8^+^ tumor-infiltrated lymphocytes (Qian et al. 2020). Another recent study reported microvesicle release in pancreatic cancer cells treated with nsPEF (Szlasa et al. 2023). However, structures shown in the micrographs in this paper were micrometer-sized, identified solely by label-free morphological imaging, and lacked direct evidence of vesicle detachment. Rather than representing shed microvesicles, they were consistent with conventional post-electroporation blebs documented extensively in the earlier literature (Michel et al. 2020; Muratori et al. 2017; Pakhomov et al. 2007; Pakhomova et al. 2013; Tekle et al. 2008; Tolstykh et al. 2017; Vincelette et al. 2013). A follow-up study (Szlasa et al. 2024) added electron microscopy images of structures interpreted as evidence of PEF-induced exocytosis in A375 melanoma cells, but without unexposed control images or statistical data.

Our study provides the first evidence linking PS externalization to vesicle shedding in PEF-treated cells. The mechanism of vesicle shedding, vesicle cargo, and its potential significance for PEF-based therapies will be prioritized in the coming studies.

## Data Availability

Data will be made available on request.

## References

[CR1] Armstrong JK, Wenby RB, Meiselman HJ, Fisher TC (2004) The hydrodynamic radii of macromolecules and their effect on red blood cell aggregation. Biophys J 87:4259–4270 15361408 10.1529/biophysj.104.047746 PMC1304934

[CR2] Back N, Kanerva K, Kurutihalli V, Yanik A, Ikonen E, Mains RE, Eipper BA (2017) The endocytic pathways of a secretory granule membrane protein in HEK293 cells: PAM and EGF traverse a dynamic multivesicular body network together. Eur J Cell Biol 96:407–417 28377049 10.1016/j.ejcb.2017.03.007 PMC5592135

[CR3] Beebe SJ, Fox PM, Rec LJ, Somers K, Stark RH, Schoenbach KH (2002) Nanosecond pulsed electric field (nsPEF) effects on cells and tissues: apoptosis induction and tumor growth inhibition. IEEE Trans Plasma Sci 30:286–292

[CR4] Beebe SJ, Blackmore PF, White J, Joshi RP, Schoenbach KH (2004) Nanosecond pulsed electric fields modulate cell function through intracellular signal transduction mechanisms. Physiol Meas 25:1077–1093 15382843 10.1088/0967-3334/25/4/023

[CR5] Bevers EM, Comfurius P, Dekkers DW, Zwaal RF (1999) Lipid translocation across the plasma membrane of mammalian cells. Biochim Biophys Acta 1439:317–330 10446420 10.1016/s1388-1981(99)00110-9

[CR6] Bhattacharya S, Silkunas M, Gudvangen E, Mangalanathan U, Pakhomova ON, Pakhomov AG (2022) Ca(2+) dependence and kinetics of cell membrane repair after electropermeabilization. Biochim Biophys Acta Biomembr 1864:183823 34838875 10.1016/j.bbamem.2021.183823

[CR7] Breton M, Mir LM (2018) Investigation of the chemical mechanisms involved in the electropulsation of membranes at the molecular level. Bioelectrochem 119:76–83 10.1016/j.bioelechem.2017.09.005 28917184

[CR8] Carman CV, Nikova DN, Sakurai Y, Shi J, Novakovic VA, Rasmussen JT, Lam WA, Gilbert GE (2023) Membrane curvature and PS localize coagulation proteins to filopodia and retraction fibers of endothelial cells. Blood Adv 7:60–72 35849711 10.1182/bloodadvances.2021006870 PMC9827038

[CR9] Cole RW, Jinadasa T, Brown CM (2011) Measuring and interpreting point spread functions to determine confocal microscope resolution and ensure quality control. Nat Protoc 6:1929–1941 22082987 10.1038/nprot.2011.407

[CR10] Cooper ST, McNeil PL (2015) Membrane repair: mechanisms and pathophysiology. Physiol Rev 95:1205–1240 26336031 10.1152/physrev.00037.2014 PMC4600952

[CR11] Copic A, Dieudonne T, Lenoir G (2023) Phosphatidylserine transport in cell life and death. Curr Opin Cell Biol 83:102192 37413778 10.1016/j.ceb.2023.102192

[CR12] Cumming G, Fidler F, Vaux DL (2007) Error bars in experimental biology. J Cell Biol 177:7–11 17420288 10.1083/jcb.200611141 PMC2064100

[CR13] Demonbreun AR, McNally EM (2016) Plasma membrane repair in health and disease. Curr Top Membr 77:67–96 26781830 10.1016/bs.ctm.2015.10.006 PMC4827257

[CR14] Flaskamp L, Prechtl M, Scheck A, Hu W, Ried C, Kislinger G, Simons M, Krug AB, Kranich J, Brocker T (2025) Assessing extracellular vesicle turnover in vivo using highly sensitive phosphatidylserine-binding reagents. Adv Sci (Weinh) 12:e07624 40817753 10.1002/advs.202507624 PMC12561326

[CR15] Hall EH, Schoenbach KH, Beebe SJ (2005) Nanosecond pulsed electric fields (nsPEF) induce direct electric field effects and biological effects on human colon carcinoma cells. DNA Cell Biol 24:283–291 15869405 10.1089/dna.2005.24.283

[CR16] Hanna H, Denzi A, Liberti M, Andre FM, Mir LM (2017) Electropermeabilization of inner and outer cell membranes with microsecond pulsed electric fields: quantitative study with calcium ions. Sci Rep 7:13079 29026094 10.1038/s41598-017-12960-w PMC5638809

[CR17] Hristov K, Mangalanathan U, Casciola M, Pakhomova ON, Pakhomov AG (2018) Expression of voltage gated calcium channels augments cell susceptibility to membrane disruption by nanosecond pulsed electric field. Biochim et Biophys Acta (BBA) Biomembr 1860:2175–2183 10.1016/j.bbamem.2018.08.017 30409513

[CR18] Hu Q, Joshi RP, Schoenbach KH (2005) Simulations of nanopore formation and phosphatidylserine externalization in lipid membranes subjected to a high-intensity, ultrashort electric pulse. Phys Rev E Stat Nonlin Soft Matter Phys 72:031902 16241477 10.1103/PhysRevE.72.031902

[CR19] Jimenez AJ, Perez F (2017) Plasma membrane repair: the adaptable cell life-insurance. Curr Opin Cell Biol 47:99–107 28511145 10.1016/j.ceb.2017.03.011

[CR20] Kay JG, Grinstein S (2011) Sensing phosphatidylserine in cellular membranes. Sensors Basel 11:1744–1755 22319379 10.3390/s110201744 PMC3274058

[CR21] Kay JG, Koivusalo M, Ma X, Wohland T, Grinstein S (2012) Phosphatidylserine dynamics in cellular membranes. Mol Biol Cell 23:2198–2212 22496416 10.1091/mbc.E11-11-0936 PMC3364182

[CR22] Kotnik T, Rems L, Tarek M, Miklavcic D (2019) Membrane electroporation and electropermeabilization: mechanisms and models. Annu Rev Biophys 48:63–91 30786231 10.1146/annurev-biophys-052118-115451

[CR23] Levine ZA, Vernier PT (2010) Life cycle of an electropore: field-dependent and field-independent steps in pore creation and annihilation. J Membr Biol 236:27–36 20623350 10.1007/s00232-010-9277-y

[CR24] Marracino P, Bernardi M, Liberti M, Del Signore F, Trapani E, Garate JA, Burnham CJ, Apollonio F, English NJ (2018) Transprotein-Electropore Characterization: A Molecular Dynamics Investigation on Human AQP4. ACS Omega 3:15361–15369 30556005 10.1021/acsomega.8b02230 PMC6288775

[CR25] Michel O, Pakhomov AG, Casciola M, Saczko J, Kulbacka J, Pakhomova ON (2020) Electropermeabilization does not correlate with plasma membrane lipid oxidation. Bioelectrochem 132:107433 10.1016/j.bioelechem.2019.107433 31891877

[CR26] Muratori C, Pakhomov AG, Gianulis E, Meads J, Casciola M, Mollica PA, Pakhomova ON (2017) Activation of the phospholipid scramblase TMEM16F by nanosecond pulsed electric fields (nsPEF) facilitates its diverse cytophysiological effects. J Biol Chem 292:19381–19391 28982976 10.1074/jbc.M117.803049 PMC5702676

[CR27] Murphy KR, Aycock KN, Marsh S, Hay AN, Athanasiadi I, Bracha S, Chang C, Gourdie R, Davalos RV, Rossmeisl JH, Dervisis NG (2024) Tumor-derived extracellular vesicles disrupt the blood-brain barrier endothelium following high-frequency irreversible electroporation. Sci Rep 14:28533 39557959 10.1038/s41598-024-79019-5 PMC11574144

[CR28] Murphy KR, Aycock KN, Marsh S, Yang L, Hinckley J, Selmek A, Gourdie R, Bracha S, Davalos RV, Rossmeisl JH, Dervisis NG (2025) High frequency irreversible electroporation alters proteomic profiles and tropism of small tumor derived extracellular vesicles to promote immune cell infiltration. Cells 14:1782 41294835 10.3390/cells14221782 PMC12651280

[CR29] Nagata S, Sakuragi T, Segawa K (2020) Flippase and scramblase for phosphatidylserine exposure. Curr Opin Immunol 62:31–38 31837595 10.1016/j.coi.2019.11.009

[CR30] Pakhomov AG, Shevin R, White JA, Kolb JF, Pakhomova ON, Joshi RP, Schoenbach KH (2007) Membrane permeabilization and cell damage by ultrashort electric field shocks. Arch Biochem Biophys 465:109–118 17555703 10.1016/j.abb.2007.05.003

[CR31] Pakhomov AG, Bowman AM, Ibey BL, Andre FM, Pakhomova ON, Schoenbach KH (2009) Lipid nanopores can form a stable, ion channel like conduction pathway in cell membrane. Biochem Biophys Res Commun 385:181–186 19450553 10.1016/j.bbrc.2009.05.035 PMC2739132

[CR32] Pakhomova ON, Gregory BW, Semenov I, Pakhomov AG (2013) Two modes of cell death caused by exposure to nanosecond pulsed electric field. PLoS ONE 8:e70278 23894630 10.1371/journal.pone.0070278 PMC3720895

[CR33] Prevc A, Bedina Zavec A, Cemazar M, Kloboves-Prevodnik V, Stimac M, Todorovic V, Strojan P, Sersa G (2016) Bystander effect induced by electroporation is possibly mediated by microvesicles and dependent on pulse amplitude, repetition frequency and cell type. J Membr Biol 249:703–711 27371159 10.1007/s00232-016-9915-0

[CR34] Qian J, Chen T, Wu Q, Zhou L, Zhou W, Wu L, Wang S, Lu J, Wang W, Li D, Xie H, Su R, Guo D, Liu Z, He N, Yin S, Zheng S (2020) Blocking exposed PD-L1 elicited by nanosecond pulsed electric field reverses dysfunction of CD8(+) T cells in liver cancer. Cancer Lett 495:1–11 32949680 10.1016/j.canlet.2020.09.015

[CR35] Rems L, Viano M, Kasimova MA, Miklavcic D, Tarek M (2019) The contribution of lipid peroxidation to membrane permeability in electropermeabilization: a molecular dynamics study. Bioelectrochem 125:46–57 10.1016/j.bioelechem.2018.07.018 30265863

[CR36] Rems L, Kasimova MA, Testa I, Delemotte L (2020) Pulsed electric fields can create pores in the voltage sensors of voltage-gated ion channels. Biophys J 119:190–205 32559411 10.1016/j.bpj.2020.05.030 PMC7335976

[CR37] Roth CC, Tolstykh GP, Payne JA, Kuipers MA, Thompson GL, DeSilva MN, Ibey BL (2013) Nanosecond pulsed electric field thresholds for nanopore formation in neural cells. J Biomed Opt 18:035005 23532338 10.1117/1.JBO.18.3.035005

[CR38] Ruiz-Fernandez AR, Campos L, Villanelo F, Gutierrez-Maldonado SE, Perez-Acle T (2021) Exploring the conformational changes induced by nanosecond pulsed electric fields on the voltage sensing domain of a Ca(2+) channel. Membranes (Basel) 11:473 34206827 10.3390/membranes11070473 PMC8303878

[CR39] Scheffer LL, Sreetama SC, Sharma N, Medikayala S, Brown KJ, Defour A, Jaiswal JK (2014) Mechanism of Ca(2)(+)-triggered ESCRT assembly and regulation of cell membrane repair. Nat Commun 5:5646 25534348 10.1038/ncomms6646 PMC4333728

[CR40] Segawa K, Nagata S (2015) An apoptotic ’eat me’ signal: phosphatidylserine exposure. Trends Cell Biol 25:639–650 26437594 10.1016/j.tcb.2015.08.003

[CR41] Semenov I, Xiao S, Pakhomova ON, Pakhomov AG (2013) Recruitment of the intracellular Ca^2+^ by ultrashort electric stimuli: the impact of pulse duration. Cell Calcium 54:145–150 23777980 10.1016/j.ceca.2013.05.008 PMC3759600

[CR42] Semenov I, Zemlin C, Pakhomova ON, Xiao S, Pakhomov AG (2015) Diffuse, non-polar electropermeabilization and reduced propidium uptake distinguish the effect of nanosecond electric pulses. Biochim Biophys Acta 1848:2118–2125 26112464 10.1016/j.bbamem.2015.06.018 PMC4554928

[CR43] Shi J, Shi Y, Waehrens LN, Rasmussen JT, Heegaard CW, Gilbert GE (2006) Lactadherin detects early phosphatidylserine exposure on immortalized leukemia cells undergoing programmed cell death. Cytometry A 69:1193–1201 17123296 10.1002/cyto.a.20345

[CR44] Silkunas M, Silkuniene G, Pakhomov AG (2023) Real-time imaging of individual electropores proves their longevity in cells. Biochem Biophys Res Commun 695:149408 38157631 10.1016/j.bbrc.2023.149408 PMC10842338

[CR45] Silkunas M, Pakhomova ON, Silkuniene G, Pakhomov AG (2024) Dynamics of cell membrane lesions and adaptive conductance under the electrical stress. Cell Stress 8:69–82 39135750 10.15698/cst2024.08.298 PMC11318148

[CR46] Silkuniene G, Mangalanathan UM, Pakhomov AG, Pakhomova ON (2023a) Silencing of ATP1A1 attenuates cell membrane disruption by nanosecond electric pulses. Biochem Biophys Res Commun 677:93–97 37566922 10.1016/j.bbrc.2023.08.011

[CR47] Silkuniene G, Mangalanathan UM, Rossi A, Mollica PA, Pakhomov AG, Pakhomova O (2023b) Identification of proteins involved in cell membrane permeabilization by nanosecond electric pulses (nsEP). Int J Mol Sci 24:9191 37298142 10.3390/ijms24119191 PMC10253066

[CR48] Szlasa W, Michel O, Sauer N, Novickij V, Lewandowski D, Kasperkiewicz P, Tarek M, Saczko J, Kulbacka J (2023) Nanosecond pulsed electric field suppresses growth and reduces multi-drug resistance effect in pancreatic cancer. Sci Rep 13:351 36611083 10.1038/s41598-023-27605-4 PMC9825384

[CR49] Szlasa W, Sauer N, Baczynska D, Zietek M, Haczkiewicz-Lesniak K, Karpinski P, Fleszar M, Fortuna P, Kulus MJ, Piotrowska A, Kmiecik A, Baranska A, Michel O, Novickij V, Tarek M, Kasperkiewicz P, Dziegiel P, Podhorska-Okolow M, Saczko J, Kulbacka J (2024) Pulsed electric field induces exocytosis and overexpression of MAGE antigens in melanoma. Sci Rep 14:12546 38822068 10.1038/s41598-024-63181-x PMC11143327

[CR50] Tarek M (2005) Membrane electroporation: a molecular dynamics simulation. Biophys J 88:4045–4053 15764667 10.1529/biophysj.104.050617 PMC1305635

[CR51] Tekle E, Wolfe MD, Oubrahim H, Chock PB (2008) Phagocytic clearance of electric field induced ’apoptosis-mimetic’ cells. Biochem Biophys Res Commun 376:256–260 18771656 10.1016/j.bbrc.2008.08.060 PMC2716758

[CR52] Tolstykh GP, Thompson GL, Beier HT, Steelman ZA, Ibey BL (2017) nsPEF-induced PIP2 depletion, PLC activity and actin cytoskeletal cortex remodeling are responsible for post-exposure cellular swelling and blebbing. Biochem Biophys Rep 9:36–41 28955986 10.1016/j.bbrep.2016.11.005 PMC5614542

[CR53] van Engeland M, Nieland LJ, Ramaekers FC, Schutte B, Reutelingsperger CP (1998) Annexin V-affinity assay: a review on an apoptosis detection system based on phosphatidylserine exposure. Cytometry 31:1–9 9450519 10.1002/(sici)1097-0320(19980101)31:1<1::aid-cyto1>3.0.co;2-r

[CR54] Vernier PT, Sun Y, Marcu L, Craft CM, Gundersen MA (2004a) Nanoelectropulse induced phosphatidylserine translocation. Biophys J 86:4040–4048 15189899 10.1529/biophysj.103.037945 PMC1304304

[CR55] Vernier PT, Sun Y, Marcu L, Craft CM, Gundersen MA (2004b) Nanosecond pulsed electric fields perturb membrane phospholipids in T lymphoblasts. FEBS Lett 572:103–108 15304332 10.1016/j.febslet.2004.07.021

[CR56] Vernier PT, Sun Y, Gundersen MA (2006a) Nanoelectropulse-driven membrane perturbation and small molecule permeabilization. BMC Cell Biol 7:37 17052354 10.1186/1471-2121-7-37 PMC1624827

[CR57] Vernier PT, Ziegler MJ, Sun Y, Gundersen MA, Tieleman DP (2006b) Nanopore-facilitated, voltage-driven phosphatidylserine translocation in lipid bilayers in cells and in silico. Phys Biol 3:233–247 17200599 10.1088/1478-3975/3/4/001

[CR58] Vincelette RL, Roth CC, McConnell MP, Payne JA, Beier HT, Ibey BL (2013) Thresholds for phosphatidylserine externalization in Chinese hamster ovarian cells following exposure to nanosecond pulsed electrical fields (nsPEF). PLoS ONE 8:e63122 23658665 10.1371/journal.pone.0063122 PMC3639203

[CR59] Waehrens LN, Heegaard CW, Gilbert GE, Rasmussen JT (2009) Bovine lactadherin as a calcium-independent imaging agent of phosphatidylserine expressed on the surface of apoptotic HeLa cells. J Histochem Cytochem 57:907–914 19546474 10.1369/jhc.2009.953729 PMC2746724

[CR60] Williams JK, Ngo JM, Lehman IM, Schekman R (2023) Annexin A6 mediates calcium-dependent exosome secretion during plasma membrane repair. Elife 12:e86556 37204294 10.7554/eLife.86556 PMC10241516

[CR61] Wu N, Cernysiov V, Davidson D, Song H, Tang J, Luo S, Lu Y, Qian J, Gyurova IE, Waggoner SN, Trinh VQ, Cayrol R, Sugiura A, McBride HM, Daudelin JF, Labrecque N, Veillette A (2020) Critical role of lipid scramblase TMEM16F in phosphatidylserine exposure and repair of plasma membrane after pore formation. Cell Rep 30:1129-1140 e5 31995754 10.1016/j.celrep.2019.12.066 PMC7104872

[CR62] Yoo M, Kim KH (2026) Phosphatidylserine externalization in cancer: biology, immune suppression, and emerging theragnostic strategies. Int J Mol Sci 27:697 41596348 10.3390/ijms27020697 PMC12841397

[CR63] Zhao J, Zhou Q, Wiedmer T, Sims PJ (1998) Level of expression of phospholipid scramblase regulates induced movement of phosphatidylserine to the cell surface. J Biol Chem 273:6603–6606 9506954 10.1074/jbc.273.12.6603

